# Validation of High Resolution Melting Analysis (HRM) of the Amplified ITS2 Region for the Detection and Identification of Yeasts from Clinical Samples: Comparison with Culture and MALDI-TOF Based Identification

**DOI:** 10.1371/journal.pone.0132149

**Published:** 2015-08-21

**Authors:** Hans Duyvejonck, Piet Cools, Johan Decruyenaere, Kristien Roelens, Lucien Noens, Stefan Vermeulen, Geert Claeys, Ellen Decat, Els Van Mechelen, Mario Vaneechoutte

**Affiliations:** 1 Laboratory Bacteriology Research, Department of Clinical Chemistry, Microbiology and Immunology, Faculty of Medicine and Health Sciences, University of Ghent, De Pintelaan 185, 9000, Ghent, Belgium; 2 Department of Intensive Care Medicine, Ghent University Hospital, De Pintelaan 185, 9000, Ghent, Belgium; 3 Department of Obstetrics and Gynaecology, Faculty of Medicine and Health Sciences, Ghent University Hospital, 9000, Ghent, Belgium; 4 Department of Haematology and Blood Bank, Ghent University Hospital, 9000, Ghent, Belgium; 5 Department of Biomedical Sciences, Faculty of Education, Health and Social Work, University College Ghent, Keramiekstraat 80, 9000, Ghent, Belgium; 6 Department of Clinical Chemistry, Microbiology and Immunology, Ghent University Hospital, De Pintelaan 185, 9000, Ghent, Belgium; Louisiana State University, UNITED STATES

## Abstract

**Aim:**

*Candida* species are known as opportunistic pathogens, and a possible cause of invasive infections. Because of their species-specific antimycotic resistance patterns, reliable techniques for their detection, quantification and identification are needed. We validated a DNA amplification method for direct detection of *Candida* spp. from clinical samples, namely the ITS2-High Resolution Melting Analysis (direct method), by comparing it with a culture and MALDI-TOF Mass Spectrometry based method (indirect method) to establish the presence of *Candida* species in three different types of clinical samples.

**Materials and Methods:**

A total of 347 clinical samples, i.e. throat swabs, rectal swabs and vaginal swabs, were collected from the gynaecology/obstetrics, intensive care and haematology wards at the Ghent University Hospital, Belgium. For the direct method, ITS2-HRM was preceded by NucliSENS easyMAG DNA extraction, directly on the clinical samples. For the indirect method, clinical samples were cultured on *Candida* ID and individual colonies were identified by MALDI-TOF.

**Results:**

For 83.9% of the samples there was complete concordance between both techniques, i.e. the same *Candida* species were detected in 31.1% of the samples or no *Candida* species were detected in 52.8% of the samples. In 16.1% of the clinical samples, discrepant results were obtained, of which only 6.01% were considered as major discrepancies. Discrepancies occurred mostly when overall numbers of *Candida* cells in the samples were low and/or when multiple species were present in the sample.

**Discussion:**

Most of the discrepancies could be decided in the advantage of the direct method. This is due to samples in which no yeast could be cultured whereas low amounts could be detected by the direct method and to samples in which high quantities of *Candida robusta* according to ITS2-HRM were missed by culture on *Candida* ID agar. It remains to be decided whether the diagnostic advantages of the direct method compensate for its disadvantages.

## Introduction

The incidence and importance of invasive fungal infection, generally associated with high morbidity and mortality rates [1], continue to increase among intensive care unit (ICU) patients, with a global average ranging between 5 and 10 cases per 1000 ICU admissions. This increasing incidence of candidemia is mainly due to the growing number of invasive methods [2,3] and the frequent use of broad-spectrum antibiotics, leading to the suppression of bacterial colonization and possible subsequent proliferation of pathogenic fungi [4,5]. In addition, effective antifungal therapy is often started with delay due to the time consuming culture based identification and susceptibility testing of fungi [2,3].

*Candida albicans* is still the most important opportunistic fungal pathogen causing infections in humans [6]. However, candidemia caused by non-*albicans Candida* species is increasing worldwide [1]. The most prevalent non-*albicans Candida* species of clinical importance include *C*. *dubliniensis*, *C*. *glabrata*, *C*. *parapsilosis*, *C*. *tropicalis* and *C*. *krusei* [7–10]. This increase in diversity and the associated species-dependent susceptibility to antimycotics [11] complicate the appropriate choice of antifungal therapy. For example, almost all *C*. *albicans* isolates are susceptible to fluconazole, a first-line antimycotic in the treatment of candidiasis. But non-*albicans* species, such as *C*. *glabrata* and *C*. *krusei*, are respectively partially or totally resistant to this antimycotic agent, with as a consequence that inappropriate therapy is continued until proper identification of the yeast becomes available. Over the past decade, echinocandins are more frequently used as first-line therapy for invasive candidiasis because of their broad-spectrum antimycotic activity. However, the emerging resistance of *C*. *glabrata* against echinocandins has been suggested to be the result of the extensive use of echinocandins [11,12].

These observations emphasize the importance of developing methods for rapid and accurate species identification [13,14], especially in patients requiring intensive care [12].

Since conventional phenotypic methods are often time-consuming and their results are sometimes ambiguous, molecular techniques for routine identification of fungi have been developed [15–22].

We previously optimized ITS2-High Resolution Melting curve analysis (ITS2-HRM) and showed that this technique, applied after ITS2-qPCR, led to the unambiguous identification of the clinically most relevant *Candida* species [23]. The use of ITS2-HRM enables elimination of the additional step of electrophoresis or sequencing, increasing the speed of the technique and rendering the assay less labor-intensive and more cost-effective compared to approaches relying on electrophoresis and/or sequencing. When applied to cultured strains, DNA extraction can be obtained by boiling colonies, a simple and inexpensive method.

The aim of this study was to validate the ability of using ITS2-HRM for the rapid detection and identification of the clinically most relevant *Candida* species, and *Saccharomyces cerevisiae*, directly from clinical samples, i.e. without prior cultivation. Whereas Decat et al. (2012) started from crude DNA extracts from cultured strains, we started from pure DNA extracts from different types of clinical samples. This more laborious and more expensive DNA extraction (combining Lyticase treatment and commercial NucliSENS easyMAG DNA extraction) was needed to remove possible inhibitory components from the clinical samples and to optimize sensitivity of the direct approach.

We assessed the applicability of ITS2-HRM directly on clinical samples by comparing it with culture of the samples (for isolation) and MALDI-TOF Mass Spectrometry (for identification) of the cultured yeasts, as used in the routine laboratory of the Ghent University Hospital.

## Materials and Methods

### Ethical approval

This procedure was approved by the Ethical Committee of the Ghent University Hospital (IRB Protocol n° 2012/703). The consent was waived by the Ethical Committee because the research did not influence diagnosis or treatment of the patients and because samples were anonymized. Hence, no written or verbal consent was obtained from the patients to use their samples. Moreover, research was carried out on rests of samples, which would have otherwise been discarded.

### Study set up

**Fig 1:**
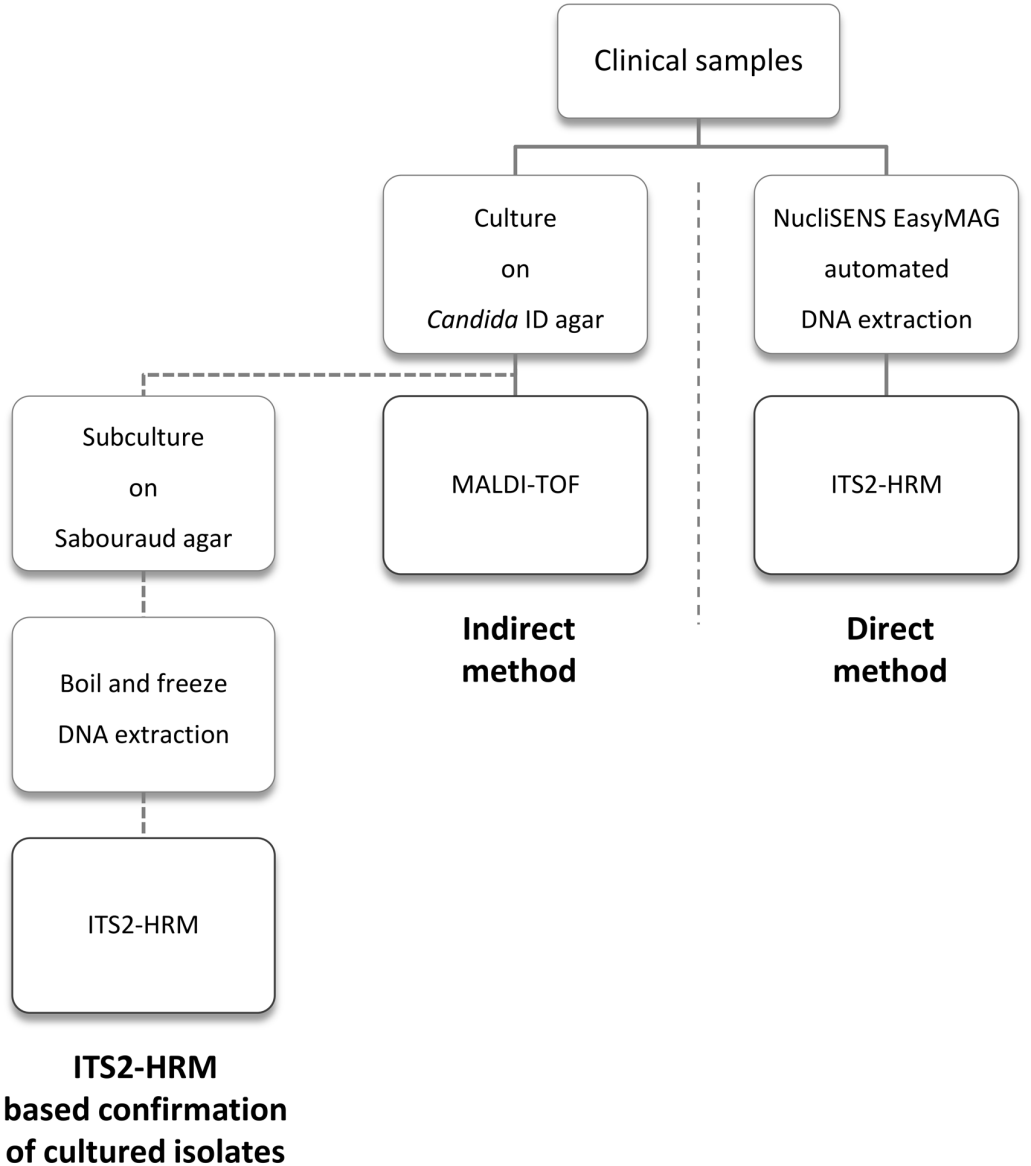
A schematic overview of the study set up.

Fig 1 summarizes the setup of the study. A total of 347 clinical samples were screened for the presence of Fungi (with emphasis on *Candida*). Once the clinical samples were collected, three different processes were followed in order to isolate and identify the *Candida* species present in the clinical samples. The indirect method, and the ITS2-HRM based confirmation method, consisted of a joint cultivation step for isolation of yeasts followed by identification, respectively with Matrix Assisted Laser Desorption Ionization—Time of Flight Mass Spectrometry (MALDI-TOF) or ITS2-HRM. In contrast to the indirect method, the direct method did not require prior culture. This method consisted of DNA extraction directly from the sample, combining Lyticase treatment and the NucliSENS easyMAG automated system, followed by the identification and quantification of the *Candida* species by means of ITS2-HRM.

### Clinical samples

Clinical samples were obtained with an ESwab. The collection tube contained 1 ml of liquid transport medium in which the entire patient sample, collected with the aid of a Nylon Flocked Swab, was delivered. The homogenous liquid sample suspension was then used for further analysis.

A total of 347 samples from three wards at the Ghent University Hospital, Belgium, were included, i.e. the gynaecology/obstetrics (n = 73), intensive care (IC, n = 200) and haematology wards (n = 74). Sample types were throat swabs (n = 111, i.e., 11 from the haematology ward and 100 from the IC), rectal swab (n = 163, i.e., 63 from the haematology ward and 100 from the IC) and vaginal swabs (n = 73, all from the gynaecology/obstetrics ward).

### Isolation of yeasts

In order to obtain isolated colonies for identification using MALDI-TOF (indirect method) and ITS2-HRM (confirmation method), 10 μl of the clinical sample was cultivated on selective ‘*Candida* ID’ medium (bioMérieux, Marcy l’Etoile, France). In the context of standardization of this study, the agar plates were incubated under the same conditions used by the routine laboratory, namely 48 hours at 32°C. Isolated colonies were further processed on Sabouraud agar (see below).

### Isolation of DNA

Two DNA extraction methods were used. For cultured yeasts, a simple boiling/freezing approach was used [23], whereas for the clinical samples, DNA was extracted by the commercial NucliSENS easyMAG extraction platform.

Boiling/freezing extraction was applied on yeast colonies subcultured on Sabouraud agar (Becton Dickinson, Erembodegem, Belgium), after initial isolation from the clinical samples on *Candida* ID agar. A 10 μl inoculation loop of yeast colonies was suspended in 250 μl TE-buffer (10 mM Tris, 100 mM EDTA, pH 8 (Sigma-Aldrich, St. Louis, MO)). This suspension was then heated during 15 minutes at 95°C, frozen for 15 minutes at -80°C, thawed at room temperature and centrifuged to pellet the cell debris. The supernatant was used as DNA extract.

Prior to the commercial DNA extraction of the clinical sample, the yeast cell wall was weakened by means of Lyticase (Sigma-Aldrich) and β-mercaptoethanol (Fluka Chemie GmbH, Buchs, Switzerland). A total of 200 μl of clinical sample was mixed with 250 μl of Lyticase Lysis Buffer (50 mM Tris-HCl (pH 8.0), 10 mM EDTA, 1.2 M sorbitol, 10 mM β-mercaptoethanol, 2 U Lyticase/μl) in 1.7 ml Eppendorf tubes, and incubated for 30 minutes at 37°C. After incubation, the suspension was transferred to 4 ml of NucliSENS easyMAG disposable sample vessels, after which 1450 μl of NucliSENS easyMAG Lysis Buffer was added. During incubation for 10 minutes at room temperature, magnetic silica was mixed in equal volumes with NucliSENS easyMAG extraction buffer 3. After the 10 min incubation period, 100 μl of magnetic silica v/v NucliSENS easyMAG extraction buffer 3 was added to each clinical sample vial (total volume of 2 ml), followed by the semi-automated NucliSENS easyMAG extraction protocol on the apparatus (bioMérieux).

### Matrix-Assisted Laser Desorption Ionization—Time of Flight

#### Mass Spectrometry

Colonies isolated from the clinical samples after 48 h of incubation at 32°C on *Candida* ID agar were identified by MALDI-TOF. Colonies with different colony morphology were spotted onto the polished steel MALDI-TOF target plate and overlaid with one μl of formic acid (Sigma-Aldrich) 70% (v/v) to weaken the cell wall of the yeast. After the plate was air dried at room temperature, one μl of matrix solution, i.e. 10 mg/ml α-cyano-4-hydroxycinnamic acid (Bruker Daltonics, Bremen, Germany) in 50% (v/v) acetonitrile/2.5% (v/v) trifluoroacetic acid (Sigma-Aldrich), was added to each spot and the plate was again air dried at room temperature. A bacterial test standard (BTS 255343, Bruker Daltonics) was used as positive control and an empty well covered with matrix served as negative control. Mass spectra were generated with a Microflex Biotyper spectrometer (Bruker Daltonics), using the manufacturer’s standard settings. For each sample, mass fingerprints were acquired, using Bruker Daltonics’ flexControl version 3.0 software, analyzed over a mass range from 2000 to 20,000 Da, and compared with the Bruker Daltonics’ database. The integrated software generated an outcome list, in which species with the most similar fingerprints were ordered according to their logarithmic score value (log (score value)) [24]. For clinical yeast isolates, log(score values) ≥ 1.7 were considered sufficient for secure species identification [25]. For values below 1.7, no isolate identification was possible [24].

#### ITS2-HRM

Each DNA extract was run in duplicate by ITS2-HRM, whereby the ITS2 region was amplified as described previously [23]. Briefly, the universal fungal primers ITS4 (5’-TCC TCC GCT TAT TGA TAT GC-3’) and ITS86 (5’-GTG AAT CAT CGA ATC TTT GAA C-3’) were used at a final concentration of 0.5 μM. Two μl of template DNA was added to a total volume of 10 μl LightCycler 480 HRM master mix (Roche, Basel, Switzerland). Amplification was carried out on a LightCycler 480 (Roche) using the following program: pre-incubation for 10 min at 95°C and amplification for 45 cycles of 20 s at 95°C, 30 s at 55°C and 30 s at 72°C, after which a high resolution melting curve was generated using the following protocol: 5 s at 95°C, 1 min at 60°C, followed by a gradual increase in temperature from 60°C to 97°C, using a ramp rate of 0.02°C per s, with one measurement per 2 s. Results were analyzed with the standard LightCycler 480 Software, version 1.5 (Roche), as described previously [23].

It should be noted that the final sample volumes used for the direct and indirect methods are not very much different between both methods: For culture, 10 μl of clinical sample is inoculated, such that, of an original presumptive 1000 cfu present in one ml of clinical sample, 10 would have been used for the culture approach.

For DNA extraction, 200 μl of clinical sample is used, which is finally eluted in 100 μl of elution buffer. Of this eluate, 2 μl is used in the qPCR, such that, of the original 1000 cfu present in one ml, 4 would have been used for the PCR approach, which is only 2.5 times less than for culture.

## Results

During validation, the results showed that there was a perfect agreement between identification with both methods, respectively MALDI-TOF and ITS2-HRM, for identification of the cultured strains. Only MALDI-TOF based identification was used to validate the direct ITS2-HRM method, because MALDI-TOF provides an independent identification approach and because this approach is used in the routine bacteriology laboratory.

The detection limit of the direct approach ranged between 1.5 cells/μl (for *C*. *dubliniensis*) and 55.6 cells/μl (for *C*. *krusei*), as determined on the basis of standard dilution series for each of the 17 species studied (Table 1). Using these standard curves of the reference strains (Table 1), optimized prior to analysis of clinical samples, quantification of the initial number of yeast cells present was possible. During each run one dilution of each of the standard series of *C*. *albicans*, *C*. *glabrata*, *C*. *dubliniensis*, *C*. *parapsilosis* and *C*. *robusta* was run along to serve as reference melting peaks for correct identification of the most prevalent species. It should be noted that quantification was not possible when two or more species were present simultaneously in the clinical sample.

**Table 1:** Overview of the detection limit and the ITS2-fragment length of the reference strains.

Species	Reference strains	GenBank accession no.	ITS2-fragment length (bp) derived from GenBank sequences	Detection limit (cells/μl)
*Candida dubliniensis*	IHEM 14280	AF218993	289	1.52
*C*. *nivariensis*	CBS 9983	KJ957824	396	1.81
*C*. *metapsilosis*	CBS 10907	KP131737	261	1.87
*C*. *parapsilosis*	ATCC 22019	AJ585347	257	2.08
*C*. *lipolytica*	CBS 6660	AF218983	190	3.36
*C*. *kefyr*	IHEM 04211	HE650694	378	5.33
*C*. *guilliermondii*	IHEM 03283	AF218996	325	5.58
*C*. *famata*	CBS 1795	AB696581	327	7.87
*C*. *lusitaniae*	IHEM 10293	AF009215	205	11.4
*C*. *albicans*	ATCC 90028	AF217609	284	12.8
*C*. *orthopsilosis*	J 960161	HG970739	262	22.8
*C*. *glabrata*	IHEM 04566	AF218994	365	23.6
*C*. *norvegensis*	CBS 6564	AF333096	319	28.6
*C*. *inconspicua*	HHR 0304 00305	KP131717	253	39.4
*C*. *tropicalis*	IHEM 04222	HG970740	274	39.5
*Saccharomyces cerevisiae* (*C*. *robusta*)	IHEM 14402	AF219006	366	40.0
*C*. *krusei*	ATCC 06258	L47113	294	55.6

Legend: ATCC: American Type Culture Collection, Rockville, Maryland; IHEM: Institute for Hygiene & Epidemiology, Mycology, Brussels, Belgium; CS Fungal Biodiversity Centre; J: non-CBS strains, obtained from CBS.

Overall, the results of both the indirect and direct methods agreed in 83.9% of samples, with 52.8% that were negative with both methods, 25.6% that were positive for the same species, 5.2% that were positive for the same two species and one sample (0.3%) positive for the same three species (Tables 2 and 3). From Table 2, sensitivity, specificity, positive predictive value (PPV) and negative predictive value (NPV) of the direct method can be calculated. A sensitivity of 91.5%, a specificity of 80.8% and a NPV of 94.9% indicate that the direct method enables to clearly distinguish between a positive and negative culture result. The direct method has a PPV of 71.1%.

**Table 2:** Comparison of the direct (ITS2-HRM) and indirect (culture/MALDI-TOF) method, expressed as percentage of samples relative to the total number (347) of samples tested.

		Indirect		Total number	
		Positive	Negative		
Direct	Positive	31.1% (TP)	12.7^a^ % (FP)	152	71.1% (PPV)
	Negative	2.9^b^ % (FN)	53.3^c^ % (TN)	195	94.9% (NPV)
Total number		118	229	347	
		91.5% (Sensitivity)	80.8% (Specificity)		

Legend: TP: True positive; FP: False-positive; FN: False-negative; TN: True negative; PPV: positive predictive value, TP/(TP+FP); NPV: negative predictive value, TN/(TN+FN); Sensitivity: TP/(TP+FN); Specificity: TN/(TN+FP)

^a^ No species or less species than the number of species detected with the direct method. This category also contains one sample that was positive only for *Achromobacter xylosoxidans* with the indirect method, while the direct method showed the presence of *Candida albicans*.

^b^ No species or less species than the number of species detected with the indirect method

^c^ Two of these samples were positive for *Achromobacter xylosoxidans* with the indirect method, but are counted as negative for Fungi.

**Table 3:** Detailed overview of the results comparing the direct and indirect method, expressed as percentages for each of 5 types of samples.

Categories	Throat (IC)	Throat (H)	Rectal (IC)	Rectal (H)	Vaginal (G)	Total
**Total number of samples**	**100**	**11**	**100**	**63**	**73**	**347**
**Negative with both methods (%)**	**25.0**	**72.7**	**55.0**	**61.9**	**76.7**	**52.8**
**Positive with both methods (1 species) (%)**	**41.0**	**9.1**	**26.0**	**12.7**	**17.8**	**25.6**
*Candida albicans*	34.0	0.0	19.0	4.8	12.3	18.7
*C*. *dubliniensis*	3.0	0.0	1.0	0.0	1.4	1.4
*C*. *glabrata*	1.0	9.1	3.0	1.6	4.1	2.6
*C*. *guilliermondii*	0.0	0.0	0.0	1.6	0.0	0.3
*C*. *parapsilosis*	2.0	0.0	0.0	3.2	0.0	1.2
*C*. *robusta*	1.0	0.0	1.0	1.6	0.0	0.9
*C*. *tropicalis*	0.0	0.0	1.0	0.0	0.0	0.3
*Geotrichum silvicola*	0.0	0.0	1.0	0.0	0.0	0.3
**Positive with both methods (2 or more species) (%)**	**12.0**	**0.0**	**6.0**	**1.6**	**0.0**	**5.5**
*C*. *albicans*/*C*. *dubliniensis*	1.0	0.0	0.0	0.0	0.0	0.3
*C*. *albicans*/*C*. *glabrata*	7.0	0.0	5.0	0.0	0.0	3.5
*C*. *glabrata*/*C*. *krusei*	1.0	0.0	1.0	0.0	0.0	0.6
*C*. *glabrata/C*. *lusitaniae*	1.0	0.0	0.0	0.0	0.0	0.3
*C*. *kefyr*/*C*. *parapsilosis*/*Pichia manshurica*	0.0	0.0	0.0	1.6	0.0	0.3
*C*. *parapsilosis*/*C*. *robusta*	2.0	0.0	0.0	0.0	0.0	0.6
**Discrepancies (%)**	**22.0**	**18.2**	**13.0**	**23.8**	**5.5**	**16.1**

Legend: IC: Intensive Care, H: Haematology, G: Gynaecology.

**Fig 2:**
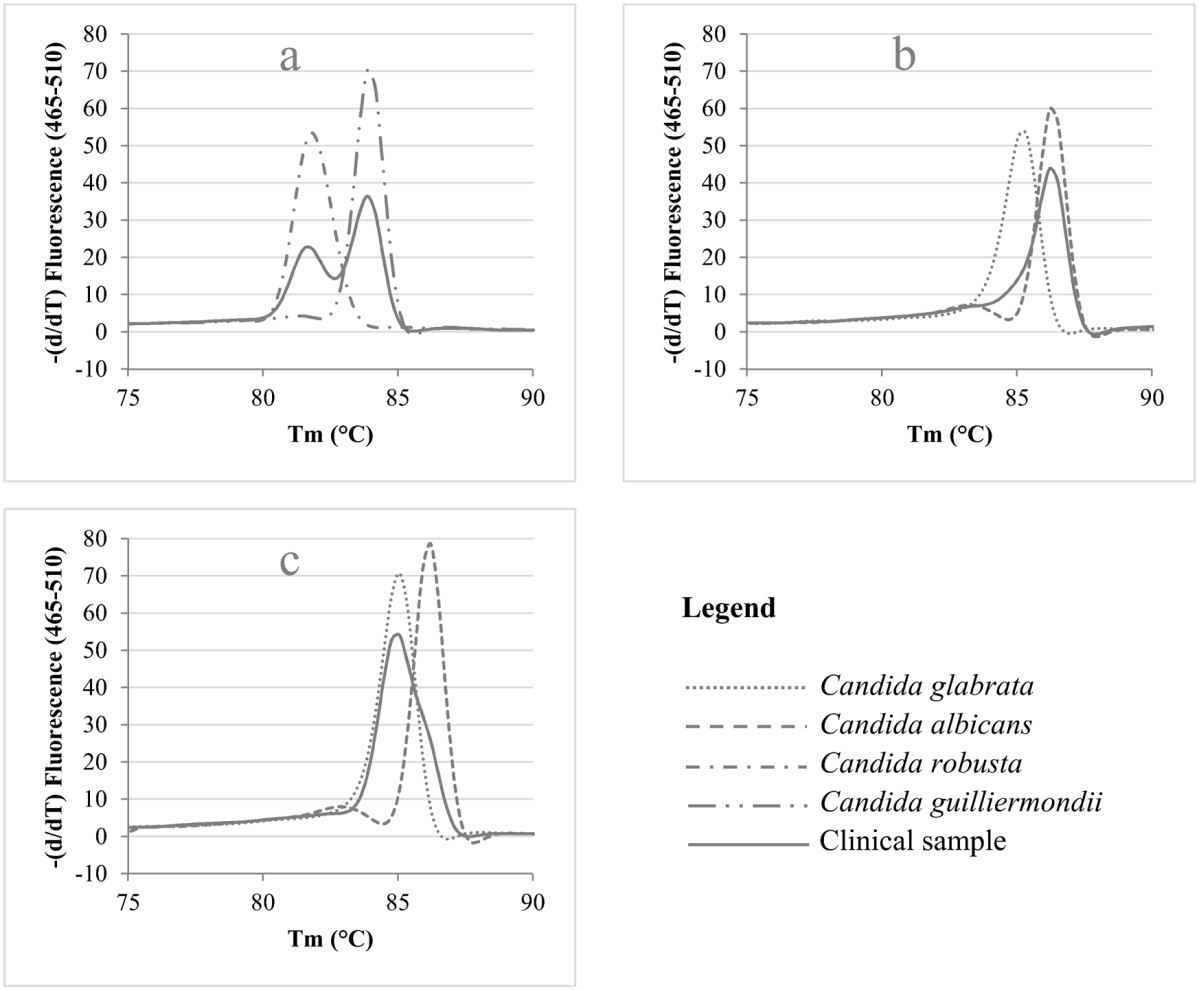
ITS2 High Resolution Melting Curve Analysis of clinical samples containing two *Candida* species. The melting curve of the sample (solid line) containing two species (according to culture results) is plotted against the melting curves of the standard strains (dotted curves) of the two respective species.

**Fig 3:**
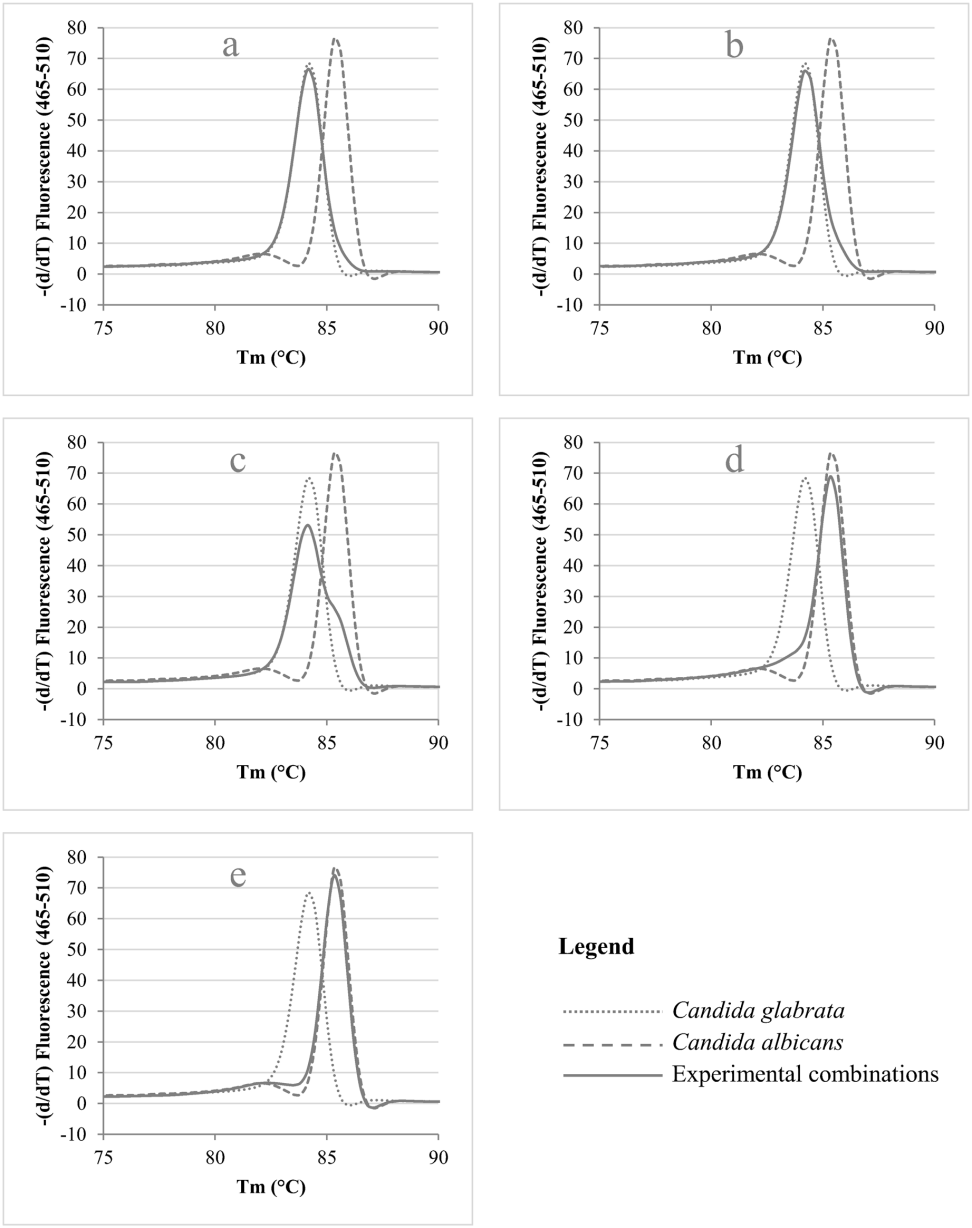
Evaluation of the impact on the shape of the melting peak pattern by analyzing 5 different experimental ratios between *Candida albicans* and *C*. *glabrata*, in which the latter one is respectively 250 (a), 200 (b), 100 (c), 10 (d) and 4 (e) fold more present.

In the cases where two or more species are present simultaneously, one would expect multiple peaks, each representing one of the species present in the clinical sample, as is the case for e.g. the combination of *C*. *guilliermondii* and *C*. *robusta* (Fig 2a). However, in the case of the simultaneous presence of *C*. *albicans* and *C*. *glabrata* (Fig 2b and 2c), only a subtle change in the melting curve of the most abundant species indicates the presence of the other *Candida* species. These findings in clinical samples led us to design an experiment in which various ratios of *C*. *albicans* and *C*. *glabrata* were mixed and analyzed with ITS2-HRM, in order to assess the impact on melting peak formation. Fig 3 illustrates the change in melting peak pattern when the ratio between *C*. *glabrata* and *C*. *albicans* is experimentally varied. It showed that *C*. *glabrata* needs to be present to a much higher degree than *C*. *albicans* in order to be observed as the main peak in the melting pattern (Fig 3a and 3b). But, even when *C*. *glabrata* is present in a higher quantity relative to *C*. *albicans*, the mean peak in the pattern still results from *C*. *albicans*. A ratio of 1 part of *C*. *albicans* to 4 parts of *C*. *glabrata* seems to be the limit in order to detect the presence of *C*. *glabrata* in a mixed sample (Fig 3e). Its presence in this situation was only indicated by the disappearance of the valley between the minor and the major peak of *Candida albicans*.

Samples with both *C*. *albicans* and *C*. *glabrata* were observed a total of 20 times, of which 17 times by the direct and 15 times by the indirect method. For 12 of these 20 samples, both the direct and the indirect method detected both species.

The most abundant species was *C*. *albicans*, present in 24.8% of all samples, and the second most abundant species was *C*. *glabrata* (9.6% of all samples). Accordingly, the most frequent type of samples containing two or more species comprised *C*. *albicans* and *C*. *glabrata*, and all of them (4.9% of all samples) were from the IC ward (Table 3).

**Fig 4:**
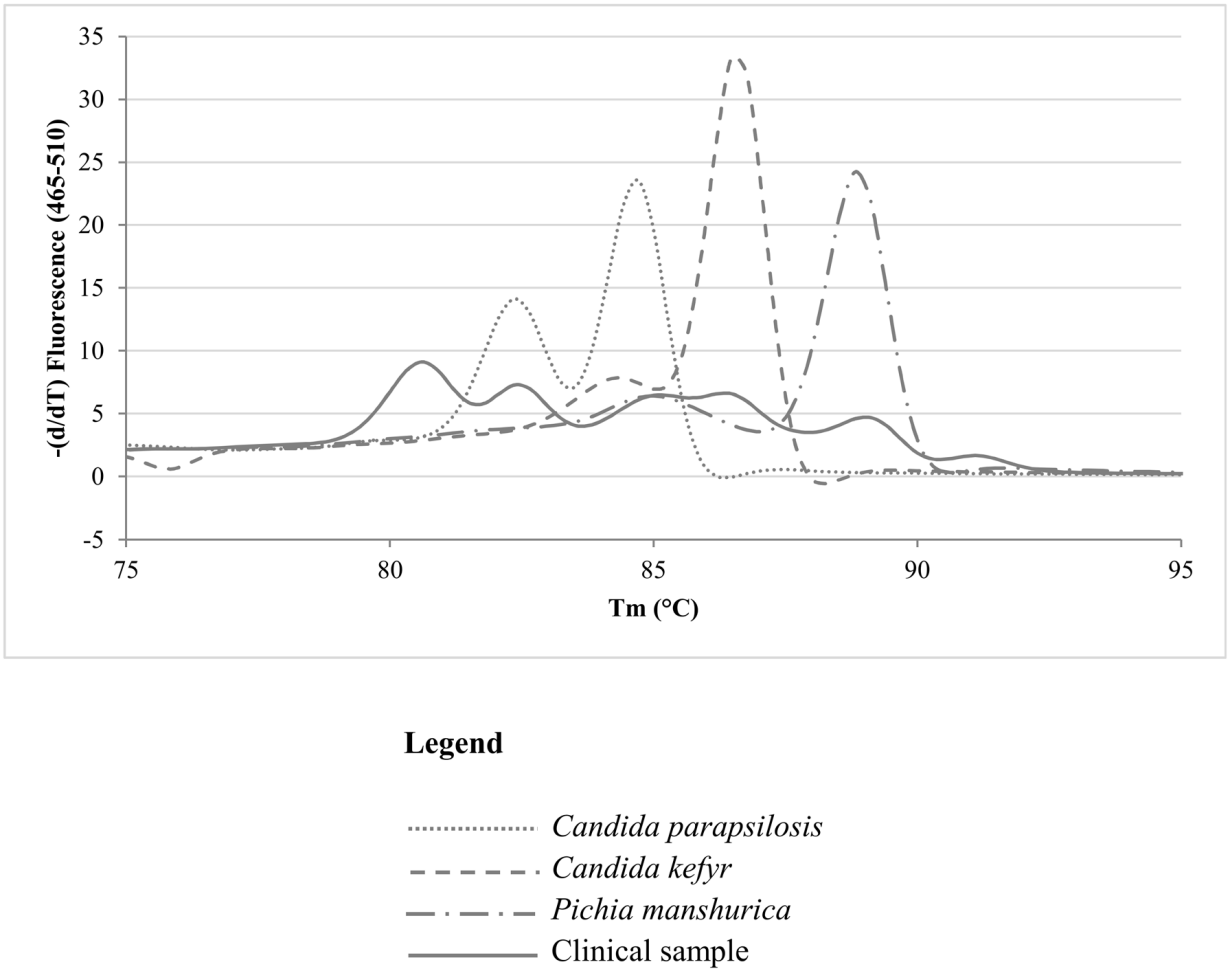
Graphic display of the ITS2-HRM curve of the sample containing *C*. *kefyr*, *C*. *parapsilosis* and *Pichia manshurica*, according to culture. ITS2-HRM patterns of strains of each species are added for comparison.

Interestingly, one sample contained *Geotrichum silvicola*, which was identified as such with culture and MALDI-TOF (indirect method) and which resulted in a melting peak of 79.7°C (direct method), not present in the *Candida* ITS2-HRM library. The same melting peak value was obtained when performing ITS2-HRM on the cultured isolate, confirming the correct identification when applying the direct method on the sample, and therefore the melting peak value of this species was added to the library. Another interesting case was presented by a sample where three different species, namely *C*. *kefyr*, *C*. *parapsilosis* and *Pichia manshurica*, were cultured and identified by MALDI-TOF. After ITS2-HRM, performed directly on the sample, a melting curve pattern (Fig 4, solid line) was obtained which comprised six low intensity melting peaks. Although the melting peaks observed in the sample corresponded to the peaks that could be obtained from the cultured isolates of the sample (Fig 4, dashed lines), direct ITS2-HRM would not have yielded a sufficiently reliable identification in this situation.

Discrepancies were observed in 56 (i.e. 16.1%) of the samples, with two samples (S01 and S54) representing two discrepancies, resulting in a total of 58 discrepancies. Most of the 58 discrepancies could be considered as minor, because in most samples whereby one method detected more species than the other, this usually concerned species that were present in low numbers. Twenty-one discrepancies were considered as major (see below), with 18 of these major discrepancies (S21-S37 and S54) in the advantage of the direct method (Table 4).

**Table 4:** Detailed overview of the discrepancies, divided into following categories: Category A: direct method positive, but indirect method negative (n = 38); Category B: direct method more species than indirect method (n = 6); Category C: indirect method positive, but direct method negative (n = 2); Category D: indirect method more species than direct method (n = 9); and Category E: aspecific detection of nonfungal species by indirect method (n = 3).

Category N°	Discrepancy	D > I ^a^	Sample ID	Culture + N°	ITS2-HRM pattern	Concentration (Cq or log cells/ml)
A+E	m+m	1	S01	ACHXYL 52	ALB	3.83
A	m	13	S02-S11, S13-S15	N	ALB	3.62–4.90
A	m	1	S12	N	ALB > GLA	Cq 28.07
A	m	1	S16	N	? ^b^	Cq 27.66
A	m	4	S17-S20	N	? ^c^	Cq 26.84–28.39
A	M	17	S21-S37	N	ROB	4.39–7.51
B	m	1	S38	ALB 1	ALB > GLA	Cq 28.08
B	m	1	S39	GLA 30	GLA > ALB	Cq 24.97
B	m	1	S40	GLA 3	GLA > ALB	Cq 29.86
B	m	1	S41	GUI 5	GUI > ROB	Cq 26.45
B	m	1	S42	GLA 4	GLA <? ^d^	Cq 26.13
B	m	1	S43	GLA 4	ALB > GLA	Cq 28.27
C	m		S44	ALB 11	N	Below detection level
D	M		S45	ALB +++/DUB +++/GLA ++	ALB	8.09
D	M		S46	ALB +++/DUB +++/GLA +++	GLA	8.60
D	M		S47	ALB +++/GLA ++	ALB	8.35
D	m		S48	ALB +++/GLA 95	ALB	7.32
D	m		S49	ALB 187/GLA 3	ALB	7.23
D	m		S50	ALB +++/LUS 1	ALB	8.09
D	m		S51	ALB 108/ROB 1	ALB	6.63
D	m		S52	PAR +++/GUI 53	PAR	8.17
D	m		S53	ROB +++/PAR 74	ROB	9.39
A+C	M+m	1	S54	TRO 2	ROB	6.83
E	m		S55-S56	ACHXYL +++	N	Below detection level

Legend: ALB: *Candida albicans*; DUB: *Candida dubliniensis*; GLA: *Candida glabrata*; GUI: *Candida guilliermondii*; LUS: *Candida lusitaniae*; PAR: *Candida parapsilosis*; ROB: *Candida robusta*; TRO: *Candida tropicalis*; ACHXYL: *Achromobacter xylosoxidans*; >: The left species is more abundant than the right species; m: minor discrepancy; M: Major discrepancy; N: No species detected, below detection level; S: Sample; D: Direct method; I: Indirect method; +: Indication of the quantity of a species, on the basis of number of colonies cultured;?: Unknown melting peak pattern.

^a^: Number of samples for which the direct method established the presence of more species than the indirect method (total number: 44).

^b^: Pattern of low intensity with unknown peak. Sequencing yielded an unreliable identification (closest match *Cladosporium cladosporioides*).

^c^: Pattern of low intensity, similar to the pattern observed with a mixture of *C*. *glabrata* and *C*. *robusta*. Sequencing yielded an unreliable identification (closest match *Physarum loratum*).

^d^: Unknown peak at a Tm of 88.78°C

Overall, the 58 discrepancies (in 56 samples) could be divided in the following 5 categories (Table 4): Category A: direct method positive, but indirect method negative (n = 38); Category B: direct method more species than indirect method (n = 6); Category C: direct method negative, but indirect method positive (n = 2); Category D: direct method less species than indirect method (n = 9); and Category E: detection of nonfungal species by indirect method (n = 3).

The direct method discovered more species than the indirect method for 44 of the 56 samples with discrepant results (Table 4, column D > I).

Minor discrepancies were observed for 15 category A samples whereby *C*. *albicans* was detected by the direct method (of which one sample (S12) also contained *C*. *glabrata* according to the direct method), but not by the indirect method, because the number of cells/ml of sample was determined by ITS2-HRM to be log 4.9 or lower in all of these samples. For the six category B samples (S38-S43), two species were detected by the direct method, compared to only one by the indirect method. The most abundant of both species (as determined on the basis of the melting peak intensities) was also detected by the indirect method in five of these six cases (whereas the least abundant was detected by the indirect method in the sixth case), although this resulted only in low numbers of colonies (between 1 and 30) for all six samples. Also these six cases can be considered as minor discrepancies because the number of yeasts as estimated by the direct method was also low, according to the high Cq values (which however could not be converted to log values, because the samples contained more than one species). Another minor discrepancy was presented by the only category C sample S44 for which only the indirect method was positive for *C*. *albicans*, but whereby only 11 colonies were cultured. For the nine category D samples (S45-S53), the direct method yielded only a single species, whereas the indirect method yielded two (n = 7) or three (n = 2) species. Six of these nine cases could be considered as minor discrepancies, because the one species that was missed by the direct method was represented by only a low number of colonies in the indirect method. In the three category E samples, the direct method remained negative (S55-S56) or yielded a low number of *C*. *albicans* (S01), but the indirect method was positive. However, MALDI-TOF indicated that in all three samples, the cultured organism was a bacterium, i.e. *Achromobacter xylosoxidans*. This was considered as a minor discrepancy.

Another five samples (category A, S16-S20) comprised unknown melting peak patterns versus negative indirect method results (i.e. negative culture). Vaginal sample S16 had low intensity melting peaks, not corresponding to any of the peaks in the library and sequencing yielded an unreliable identification (See below). The other four samples (S17-S20) had a combination of peaks that was similar to the peaks observed when both *C*. *glabrata* and *C*. *robusta* were present in the sample, but sequencing yielded no identification (due to mixed sequences). The rather high Cq values indicated that the number of fungal cells present in all five of these samples was rather low, and was therefore considered a minor discrepancy.

The 21 major discrepancies comprised 18 category A samples (S21-S37: 14 rectal swabs and four throat swabs, 13 from the haematology ward, five from the IC, and S54 (rectal swab, hematology ward)), whereby the direct method detected the presence of *C*. *robusta* in relatively high loads (between log4.39 and log7.51 cells/ml), whereas this species was missed by the indirect method because the culture remained negative. For sample S54, culture yielded only two colonies of *C*. *tropicalis*. The identification of *C*. *robusta* based on the melting peak temperature was confirmed for 12 cases by sequencing of the amplified ITS2 region. Apparently, *C*. *robusta* (the anamorph of *Saccharomyces cerevisiae*), predominant in haematology samples (13 out of 74, compared to 5 out of 200 IC samples), is difficult to culture on *Candida* ID agar.

Major discrepancies were also present for three category D samples, whereby the indirect method detected *C*. *albicans* and *C*. *glabrata* in large numbers, and in two of these three samples also *C*. *dubliniensis*, whereas the direct method indicated the presence of only *C*. *albicans* (S45, S47) or only *C*. *glabrata* (S46).

In summary, there was full agreement in 83.90% of the 347 samples, minor discrepancy in 10.09% and major discrepancy in 6.01%, with most of these major discrepancies (18/21) due to the lack of detection of *C*. *robusta* (*S*. *cerevisiae*) by the culture-based method (indirect method).

The number of discrepancies for Haematology and IC departments and for throat and rectal swabs were comparable (Tables 3 and 4). Only 5.5% (4 samples) of the 73 vaginal samples resulted in outcomes that were discrepant between both methods, moreover considerable as minor discrepancies. In three of these vaginal samples (S13-S15), *C*. *albicans* was found to be present only by the direct method, but only in low amounts (between log3.95 and log4.28 cells/ml). In one vaginal sample (S16), the direct method established, at only a low concentration (Cq = 27.66), a low intensity melting peak pattern (not observed in any of the species present in the library). Sequencing of the amplified ITS2 yielded only query cover of 62% to *Cladosporium cladosporoides*.

## Discussion

In this study, the efficacy of a molecular method (designated the ‘direct method’) to quantify and identify the yeast species present in three types of clinical samples, i.e. throat swabs, rectal and vaginal swabs, from three different departments was compared with the routine method (the ‘indirect method’).

When the indirect method, i.e. culture of samples on *Candida* ID agar, followed by identification of the colonies by means of MALDI-TOF, is taken as the reference method, the direct method, consisting of DNA-extraction of the sample, followed by ITS2-qPCR and ITS2-HRM, has a low positive predictive value. However, closer inspection of the observed discrepancies (Table 4) indicates that most can be considered as minor. These minor discrepancies mostly concern samples with low numbers of yeasts, as indicated by the high Ct-values of the qPCR, whereby no yeasts could be cultured. Moreover, 18 of the 21 major discrepancies can be considered in the advantage of the direct method as well, because these concern samples that are—strongly—positive for *Candida robusta* (the anamorph of *Saccharomyces cerevisiae*) by means of the direct method, but negative by the indirect method, indicating that this species is missed by culture on *Candida* ID agar. In intensive care unit patients, the probiotic use of *Saccharomyces cerevisiae* has been documented to potentially lead to a fatal systemic disease [26–28]. The ability of the direct method to detect and quantify the presence of this species might be considered as an additional advantage. Still, to control for false positive results due to contamination from environment or during the DNA extraction procedure, simultaneous DNA extraction from sterile water should be performed.

The most abundant species was *C*. *albicans*, present in 30.6% of all 111 throat swabs, 13.5% of all 163 rectal swabs and 12.3% of all 73 vaginal samples. The second most abundant species was *C*. *glabrata* in 2.6% of samples, most abundant in throat swabs of the haematology ward (9.1% of all throat samples) and vaginal samples (4.1% of all vaginal samples). Accordingly, the most frequent type of double positive samples comprised *C*. *albicans* and *C*. *glabrata*, and all of them (3.5% of all samples) were from the IC department, equally distributed between throat and rectal swabs. Consequently, given the predominance of *C*. *albicans* and *C*. *glabrata* (and *C*. *robusta*), the development of qPCR formats, specific for these species, instead of ITS2-HRM, could be considered, to optimize the discriminative detection and quantification for samples where both species are present.

The samples containing species such as *Geotrichum silvicola* and *Pichia manshurica* indicate that ITS2-HRM also enables the detection of species of which no representative melting curves were present in the original library. These species can be recognized by carrying out ITS2-HRM on the cultured isolates that were identified by other means (e.g. MALDI-TOF) or—in case of lack of a positive culture—by sequencing the ITS2 region that was amplified from the sample. However, the latter approach is not possible when more than one species is present in the sample.

The presence of yeast in a clinical sample was also examined through gram staining. Results (data not presented) showed a correlation between the threshold cycle (Cq) and the detection of yeast through microscopical analysis. The detection was possible in samples with a Cq lower than 22, i.e. a yeast concentration of log6. Apparently, yeast present in concentrations below log6 cfu/ml will be missed by Gram stain.

In conclusion, the direct method has some advantages over the indirect method, such as i) speed (result possible within one day), ii) the possibility for more accurate quantification of the number of yeasts present in the sample (by quantitative PCR), and iii) better sensitivity by detecting also yeasts when present in low numbers, missed by culture, and by detection of *Candida robusta* (missed by culture, also when present in large numbers). It remains to be decided whether these putative advantages compensate for the disadvantages of this method, such as the cost for consumables (especially due to the need for pure DNA-extraction when handling clinical samples), estimated to amount to five times that of culture in combination with MALDI-TOF, its laboriousness, and the lack of a cultured organism, needed to assess the antimycotic susceptibility. However, the possibility offered by a molecular approach for rapid detection, quantification and identification of yeasts from blood samples would certainly compensate for the disadvantages of this approach.
