# Correction: Validation of High Resolution Melting Analysis (HRM) of the Amplified ITS2 Region for the Detection and Identification of Yeasts from Clinical Samples: Comparison with Culture and MALDI-TOF Based Identification

**DOI:** 10.1371/journal.pone.0139501

**Published:** 2015-09-25

**Authors:** Hans Duyvejonck, Piet Cools, Johan Decruyenaere, Kristien Roelens, Lucien Noens, Stefan Vermeulen, Geert Claeys, Ellen Decat, Els Van Mechelen, Mario Vaneechoutte

In the Materials and Methods section, Mass Spectrometry and ITS2-HRM should not be subsections of the Matrix-Assisted Laser Desorption Ionization–Time of Flight section. Matrix-Assisted Laser Desorption Ionization–Time of Flight Mass Spectrometry and ITS2-HRM should both be their own subsections of the Materials and Methods section.
